# Turning over New Leaves

**Published:** 1888-11-24

**Authors:** 


### Turning Over New Leaves.

### Massage.*

Dr. Tibbits strikes the right nail on the head at the very beginning of his book on "Massage and Allied Methods of Treatment," in insisting that massage must be used only under medical direction. "It is strongly insisted on," he says, "that our certificate is strictly limited to authorising the holder to carry out treatment only under the guidance of the ordinary medical attendant of the patient. We absolutely set our face against any masseur or masseuse using our certificates as an authority to treat a patient without medical sanction." This is just as it should be, and is a useful warning to all amateur masseuses—we say *amateur* advisedly, for of course no nurse who has a right to call herself *trained* would dream of prescribing or practising massage on her own responsibility.

After referring very briefly to the antiquity of massage, of which an unscientific form existed amongst the Greeks, Romans, Egyptians, Chinese, and the Arabs, who have transmitted it to us, Dr. Tibbits gives what he modestly calls a misty definition of it as the "art and science of medical rubbing, combined with various manipulations and exercises by the operator, upon the whole or part of the body of a patient, added to certain other exercises executed by the patient, and to certain allied methods of purely local application made by the operator." This is a fairly comprehensive definition of an art which cannot be taught by print, and can only be acquired by long and constant practice; nor does Dr. Tibbits profess to do more than lay down the principles. Dr. Tibbits, at the outset, pays a graceful tribute to Dr. Weir Mitchell, whom he calls the father of modern massage, and of whose book he says, "All modern books on massage are really copies, with much padding, and useless, irrelevant matter."

We are glad that Dr. Tibbits disapproves, at least in principle (for, as he afterwards uses them, they are apparently indispensable), of such alarming terms as "friction," "effleurage," "tapotement," "petrissage"—terms calculated to instil terror into the heart of a nervous patient—and even the English equivalents are no improvement, viz., "rolling," "sawing," "felling," "chopping," "spanning," "stroking," etc.

Dr. Tibbits goes on to say that, granted massage is necessary for a case, the next point to decide is what amount is required for the patient; and as this cannot be prescribed by the medical attendant, but rests entirely with the masseuse, she must use all the intelligence she is lucky enough to possess, and all the conscientiousness and thoroughness which she ought to possess if she is worthy to be called "trained," and never sink so low as to shirk doing her very best for a case because it is difficult, and takes a great deal out of her. We know that to give out the same amount of massage grip will sometimes be easier, sometimes more difficult, according to the state of the masseuse's own health; but still there need be no deviation from the rule—to do the best of which she is capable. Dr. Tibbits lays down two or three general rules: (1) To always begin with a low power, and work up to what is required; (2) not to rub too rapidly, so as to allow the communication of sensation; (3) Not to rub roughly and hurt the patient—there can be no excuse for doing so, and any part which is at first very sensitive must be carefully dealt with, and will ultimately be able to bear the full power necessary, if accustomed to it by degrees.

After these preliminary remarks, Dr. Tibbits proceeds to describe the different processes, though he is careful to explain that he does not pretend to give precise definitions.

"Friction" may be light, moderate, or strong. The first has a soothing effect, and is useful in cases of nervous headache or wakefulness. Moderate friction is more stimulating, and transmits warmth by increasing the circulation, whilst strong friction may even cause bruising and rupturing some of the small capillaries, and of course the masseuse must remember that some patients will bruise more easily than others. "Effleurage" is defined as tickling or lightly squeezing or rolling the superficial layer of muscle, and is dismissed shortly as of "little value except as a relief to masseuse and patient." "Tapotement," or "percussion," is explained to be "striking with different degrees of force," for which either the hand or an instrument may be used—the "instrument" being a sheep's bladder filled with air, and having a string or short cane attached; or an india-rubber ball filled with air; or a ball stuffed with wool and covered with washleather, each with cane attached; or else a bundle of birch branches damped with water. But though any of these may be used, the author promptly adds that very rarely will a skilful masseuse require anything more than the hand, different parts of which are used according to the amount of force needed.

The most importance is assigned to the process called "petrissage," or "kneading," which is described as a complete kneading with both hands of the muscles and organs to be operated upon, very much resembling the way a baker treats his dough, and can only really be acquired by practice. With regard to the massage of joints, in addition to these processes certain exercises such as flexion, traction, etc., peculiar to joints, must be used, and the masseuse is strongly recommended to get very explicit directions before undertaking joint cases. To make this part of his book quite complete Dr. Tibbits quotes Dr. Schreider's directions, which are also accompanied by illustrations, and show the German system. The first process spoken of is "pressure," which is done by one, two, or three fingers, according to circumstances, and admits of three modifications, such as slight, lateral, or rotatory movement; continuous movement upwards or downwards from the original starting-point; and short continuous movements of the fingers, with alternately increased and decreased pressure. This of course is a modification of "kneading." "Thrusting" is performed with the rigidly-extended hand, the finger-tips being used, or else the clenched fist, and is useful for reaching the deeper muscles, as in cases of rheumatism or neuralgia. "Hacking" is done with the edge of the hand, or with the fingers, according to the power required. "Pinching" is also a process used by Schreiber, and consists in taking up the skin as between a pair of pliers, or "with the balls of the last joints," and this is one of the most trying processes to the masseuse. Another modification of "pinching" is "squeezing," in which the fingers remain fixed, and the thumb only acts. Squeezing should at first be applied perpendicularly to the spot manipulated, and afterwards the thumb may be rubbed backwards and forwards with considerable force. Dr. Tibbits finishes up with an account of a complete massage of the whole body, in which all the foregoing processes are illustrated; though he wishes it to be clearly understood that it is quite unnecessary for a masseuse to use them all, though she should know them all thoroughly. The Continental plan is to begin at the foot and work upwards, because in many cases there may be weakness in the circulation, and upward pressure will assist the return of blood to the heart; however, there is much divergence of opinion on this point, and it cannot be deemed of vital importance.

The remainder of the volume consists of a short account of medical gymnastics, Ling's system, or the Swedish movement; different kinds of baths; electrisation, and numerous examples of the results—sometimes truly marvellous—of these different modes of allied treament; and last of all, as a postscriptum, a summary of the main facts of physiology. We can recommend Dr. Tibbits' "Massage and Allied Methods of Treatment," both to the trained masseuse as a book of reference, and to the would-be student of the art.

* "Massage and Allied Methods of Treatment. An Abstract of Lectures Delivered to Trained Nurses and Masseuses at the School of Electricity and Massage." By Herbert Tibbits, M.D. (Churchill and Co.)

